# High Fat Diet Administration during Specific Periods of Pregnancy Alters Maternal Fatty Acid Profiles in the Near-Term Rat

**DOI:** 10.3390/nu8010025

**Published:** 2016-01-04

**Authors:** Marlon E. Cerf, Emilio Herrera

**Affiliations:** 1 Diabetes Discovery Platform, South African Medical Research Council, PO Box 19070, Tygerberg, Cape Town 7505, South Africa; 2 Department of Chemistry and Biochemistry, University of San Pablo-CEU, Ctra. Boadilla del Monte km 5.3, Madrid 28668, Spain; eherrera@ceu.es

**Keywords:** docosahexaenoic acid, feto-placental, lipids, ω-3 fatty acids, ω-6 fatty acids, triglycerides

## Abstract

Excessive fat intake is a global health concern as women of childbearing age increasingly ingest high fat diets (HFDs). We therefore determined the maternal fatty acid (FA) profiles in metabolic organs after HFD administration during specific periods of gestation. Rats were fed a HFD for the first (HF1), second (HF2), or third (HF3) week, or for all three weeks (HFG) of gestation. Total maternal plasma non-esterified fatty acid (NEFA) concentrations were monitored throughout pregnancy. At day 20 of gestation, maternal plasma, liver, adipose tissue, and placenta FA profiles were determined. In HF3 mothers, plasma myristic and stearic acid concentrations were elevated, whereas docosahexaenoic acid (DHA) was reduced in both HF3 and HFG mothers. In HF3 and HFG mothers, hepatic stearic and oleic acid proportions were elevated; conversely, DHA and linoleic acid (LA) proportions were reduced. In adipose tissue, myristic acid was elevated, whereas DHA and LA proportions were reduced in all mothers. Further, adipose tissue stearic acid proportions were elevated in HF2, HF3, and HFG mothers; with oleic acid increased in HF1 and HFG mothers. In HF3 and HFG mothers, placental neutral myristic acid proportions were elevated, whereas DHA was reduced. Further, placental phospholipid DHA proportions were reduced in HF3 and HFG mothers. Maintenance on a diet, high in saturated fat, but low in DHA and LA proportions, during late or throughout gestation, perpetuated reduced DHA across metabolic organs that adapt during pregnancy. Therefore a diet, with normal DHA proportions during gestation, may be important for balancing maternal FA status.

## 1. Introduction

Fatty acids (FAs) are structural components of organs, energy sources, precursors of bioactive compounds such as eicosanoids, including prostacyclins, prostaglandins, thromboxanes, and leukotrienes. FAs also regulate the expression of transcription factors. All FAs provide energy, whereas polyunsaturated fatty acids (PUFAs) are required for structural and metabolic functions.

The ω-3 and ω-6 PUFA families are synthesized from their essential fatty acids (EFAs), namely α-linolenic acid (αLA, 18:3 ω-3) and linoleic acid (LA, 18:2 ω-6), respectively. αLA and LA cannot be synthesized *de novo* [1] and are, therefore, supplied in the diet [2]. During pregnancy, the EFAs and PUFAs cross the placenta to maintain their supply to the fetus [3]. LA is abundant in the Western dietary pattern and is the precursor of arachidonic acid (AA, 20:4 ω-6) [2]. αLA is abundant in seed oils and is the precursor of eicosapentanoic acid (EPA, 20:5 ω-3) and docosahexaenoic acid (DHA, 22:6 ω-3) [1]. Metabolically important PUFAs during development are AA, EPA, and DHA which are not required from the maternal diet during pregnancy to meet fetal demands since they can be synthesized endogenously from the EFAs. However, the conversion of EFAs to PUFAs by the fetus is very limited and, therefore, the plasma and tissue concentrations of these FAs depend mainly on exogenous supply. Thus, during periods of rapid intrauterine growth, the production of the EPAs, DHA, and AA may be inadequate and considered as EFAs for the fetuses [4]. A sufficient supply of these FAs during pregnancy and the neonatal period is critical for normal fetal growth and proper neurological development and function [5,6].

Maternal dietary FAs, particularly PUFAs, may influence epigenetic gene regulation by inducing or repressing transcription of specific genes during critical ontogenic periods [7,8] with long-term consequences for offspring health. During pregnancy, the mother transitions through different metabolic conditions, shifting from anabolism during the first two thirds of gestation, by accumulating fat depots, to a catabolic state during the last third, when fat depot breakdown is enhanced [9,10,11]. These metabolic transitions contribute to the development of maternal hyperlipidemia with major implications for fetal growth [12]. Moreover, these adaptations are altered when the maternal diet is unbalanced by a high fat (HF) content with consequences in placental nutrient transport and fetal growth [13]. Specific placental nutrient transporters, namely glucose transporter (GLUT) 1 and sodium-coupled neutral amino acid transporter (SNAT) 2, are up-regulated in response to a high fat diet (HFD) [13].

The ingestion of a HFD during pregnancy influences both maternal and offspring health outcomes as gestational HF feeding are associated with derangements in both their metabolism and physiology that may be immediate, transient or permanent. Whereas most studies focus on developmental programming effects, the present study will investigate the differential FA profiles in maternal metabolic organs near term. With the increasing proportion of women of childbearing age that are overweight or obese and consume unhealthy diets during pregnancy, there is a great need to study dietary patterns as they influence both maternal and offspring health outcomes. Since altered dietary composition during specific periods of pregnancy could differentially affect maternal FA profiles with consequences in their availability to the fetus, this study investigates the effect of a HFD administered to pregnant rats at different stages of pregnancy on lipid profiles in near-term mothers. Maternal plasma, hepatic, adipose tissue, and placenta FA profiles were determined.

## 2. Experimental Section

### 2.1. Experimental Design

Ethical approval was obtained by the Animal Ethics Committee of the South African Medical Research Council prior to experimentation. Adult female Wistar rats from our animal facility were maintained at 22 ± 2 °C, 55% ± 10% relative humidity and 12 h light/dark cycles and fed the control diet. The female rats fed a control diet were mated with age-matched male rats. After mating was confirmed by the presence of vaginal plug(s), pregnant rats were randomly assigned to the experimental groups, housed singly, and subjected to the experimental design summarized in Table 1. Briefly, pregnant mothers (*n* = 4–6 per group) were fed a control or HF diet *ad libitum* during specific periods of gestation. The HFD was formulated by in-house dieticians and was constituted by 40% fat, 14% protein, and 46% carbohydrate, whereas the control diet was acquired commercially (Epol, Pietermaritzburg, South Africa) and contained 10% fat, 15% protein, and 75% carbohydrate (Table S1). As summarized in Table 1, the groups were mothers maintained on a HFD during the first (HF1), second (HF2), or third (HF3) week of gestation, or for all three (HFG) weeks of gestation; for the remainder of gestation, mothers were fed the control diet.

**Table 1:** Experimental design.

Groups	Gestational Diet		
	**Week 1**	**Week 2**	**Week 3**
**Control**	Control	Control	Control
**HF1**	HFD	Control	Control
**HF2**	Control	HFD	Control
**HF3**	Control	Control	HFD
**HFG**	HFD	HFD	HFD

Control mothers (*n* = 4) were maintained on a standard laboratory (control) diet throughout. Experimental mothers (*n* = 4 per group, but *n* = 6 for HF2) were maintained on a high fat diet (HFD) for specific weeks of gestation. HF1, mothers maintained on a HFD for week one; HF2, mothers maintained on a HFD for week two; HF3, mothers maintained on a HFD for week three; HFG, mothers maintained on a HFD for all three weeks of gestation.

### 2.2. Sample Collection

At days zero (e0, just prior to mating), seven (e7, early pregnancy), 14 (e14, mid pregnancy) and 20 (e20, near-term), blood was collected from the tail after 3 h of fasting. Mothers were anesthetized via an anesthetic machine (Motivus Resuscitator Type AV, Crest Healthcare Technology Ltd., Johannesburg, South Africa) with fluothane (Halothane, AstraZeneca Pharmaceuticals, Johannesburg, South Africa) and 2% oxygen. After the mothers were anesthetized, the tip of the tail was heated with a ultraviolet (UV) lamp to facilitate blood flow then snipped with a surgical blade. Blood was collected in ice-cooled tubes containing 1 mg/ml of Na_2_-EDTA. After centrifugation, plasma samples were stored at −20 °C until analysis. After the last blood collection (e20), mothers were maintained under anesthesia and whole liver, lumbar adipose tissue, and placenta were collected and stored at −80 °C until analysis.

### 2.3. Determination of Blood Glucose and Serum Insulin Concentrations and Lipid Profiles

Blood glucose (glucometer, Precision QID, MediSense, Cambridge, UK) and serum insulin (rat insulin RIA kit, Linco Research, St. Charles, MO, USA) concentrations were measured. Homeostasis model assessment (HOMA)-insulin resistance ((fasting plasma glucose (mmol/L) X fasting serum insulin (mU/L))/22.5)) was calculated. Plasma triglycerides (TAG), cholesterol (Spinreact Reactives, Girona, Spain), and non-esterified fatty acid (NEFA) (Wako Chemicals, Neuss, Germany) were determined enzymatically using commercial kits. For FA profile analyses, nonadecenoic acid (19:1) (Sigma-Aldrich, Madrid, Spain) was added as the internal standard to fresh aliquots of diets and frozen plasma and tissue which were used for lipid extraction and purification [14]. For the placenta, lipid extracts were evaporated to dryness and resuspended in chloroform in the presence of activated silicic acid to extract the neutral lipids whereas the dried chloroform-washed silicic acid fraction was treated with methanol to extract the phospholipids. The final lipid extracts were evaporated under vacuum and the residue suspended in methanol/toluene followed by methanolysis in the presence of acetyl chloride at −80 °C for 2.5 h as previously described [15]. FA methyl esters were separated and quantified on a Perkin-Elmer gas chromatograph (Autosystem, Madrid, Spain) with a flame ionization detector and a 20 m Omegawax capillary column (internal diameter 0.25 mm). Nitrogen was used as the carrier gas and the FA methyl esters were compared to purified standards (Sigma-Aldrich). Quantification of the FAs in the samples was performed as a function of the corresponding peak areas and compared to the internal standard.

### 2.4. Statistical Analysis

One-way analysis of variance (ANOVA) and Bonferroni’s post-test were applied with data reported as means ± standard error of the mean (SEM) and significance established at *p* < 0.05. However, for dietary FA and fetal-maternal FA analyses, the Student’s *t*-test was applied.

## 3. Results

### 3.1. Anthropometry

Although HFG mothers consumed more food compared to the other mothers, there were no differences in body weights (Table S2). Despite no differences in placental weight, when adjusted for body weight, placental weights were reduced in HF3 mothers relative to control mothers (Table S2). There were no differences in conceptus weight, litter sizes, or any of the maternal organs that were studied (Table S2).

### 3.2. Glycemia, Insulinemia and HOMA-Insulin Resistance

There were no differences in glycemia, insulinemia, and HOMA-insulin resistance amongst the groups (Table S3).

### 3.3. Fatty Acid Profile in the Diets

**Figure 1:**
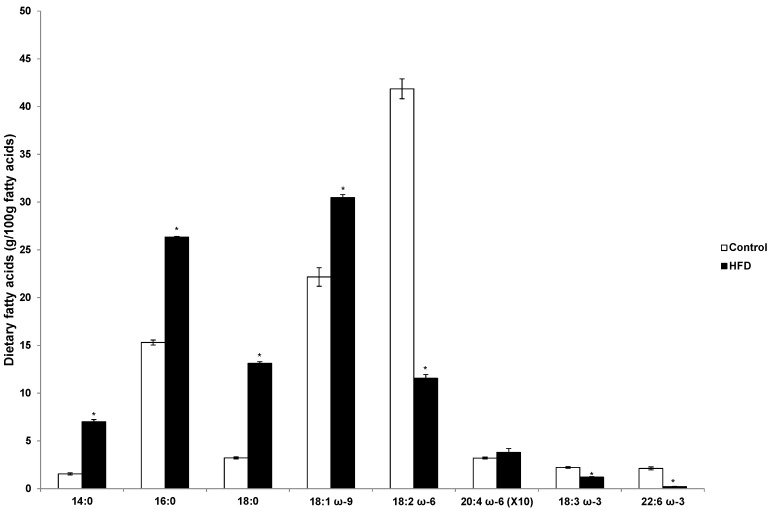
Dietary fatty acids (g/100 g fatty acids). Values are means ± standard error of the mean (SEM). HFD, high fat diet. * *p* < 0.0001.

As shown in Figure 1, the proportion of saturated FAs (namely myristic, palmitic, and stearic acids) and oleic acid were higher in the HFD than in the control diet with lower proportions of αLA, DHA, and LA in the HFD. In the HFD, palmitic and oleic acid were the predominant FAs (57% of total diet), whereas in the control diet LA was most abundant.

### 3.4. Plasma Total Non-Esterified Fatty Acid, Triglyceride, and Cholesterol Concentrations and Individual Fatty Acid Profiles

**Figure 2:**
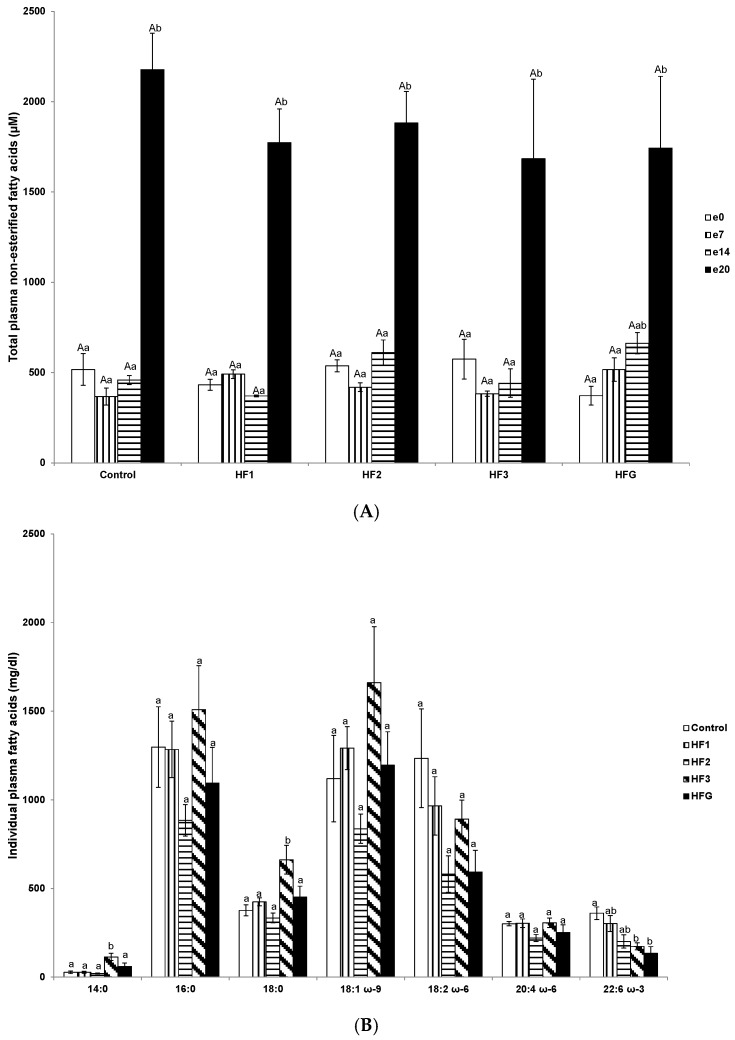

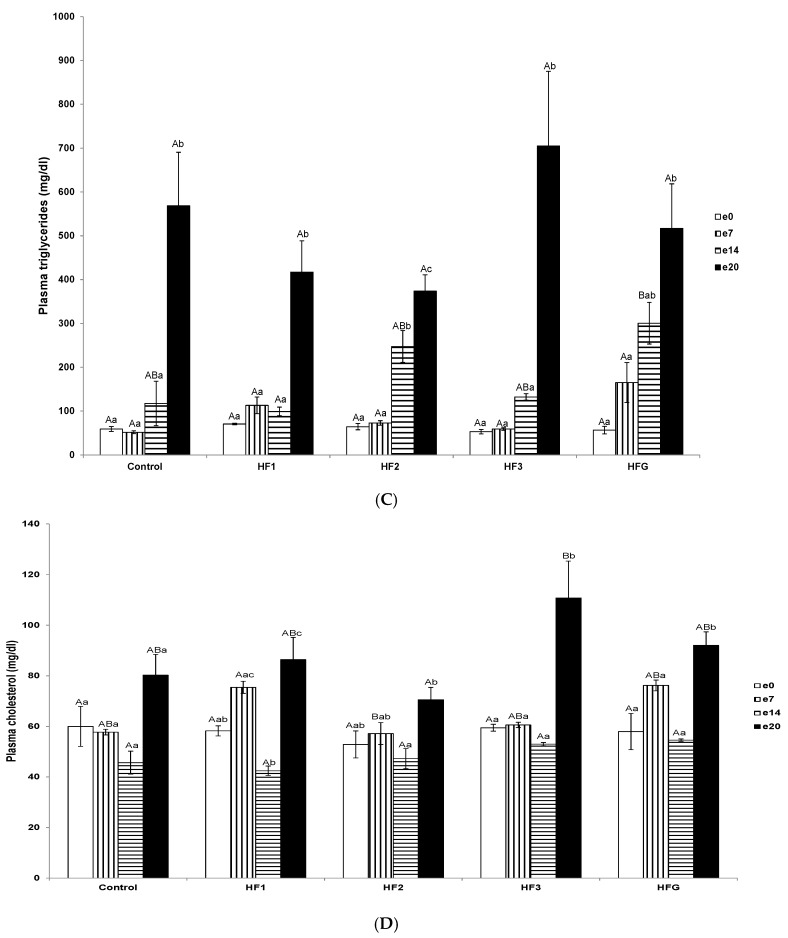
Lipid profiles during gestation. (**A**) Plasma non-esterified fatty acid concentrations (μM); (**B**) individual plasma fatty acid concentrations (mg/dL); (**C**) plasma triglyceride concentrations (mg/dL); and (**D**) plasma cholesterol concentrations (mg/dL). Numerals refer to the specific week of gestational high fat (HF) maintenance. G, gestation refers to HF maintenance throughout gestation. Values are means ± standard error of the mean (SEM). Capital letters refer to inter-groups. Lower case letters refer to intra-groups. Different letters reflect significant changes.

Near-term (e20) plasma total NEFA concentrations were elevated in control, HF1, HF2, and HF3 mothers compared to pre-gravidity (e0), early (e7), and mid-term (e14); and in HFG mothers at near-term (e20), compared to pre-gravidity (e0) and early term (e7) (Figure 2A).

With reference to the individual FA profiles at e20 in HF3 mothers, myristic (14:0) and stearic acid (18:0) concentrations were elevated compared to control, HF1, HF2, and HFG mothers (Figure 2B). However, DHA concentrations were reduced in HF3 and HFG mothers compared to the control mothers (Figure 2B). Plasma αLA values in all the groups were negligible (data not shown). 

Plasma TAG concentrations at mid-term (e14) were elevated in HFG mothers compared to HF1 mothers (Figure 2C). Further, mid-term (e14) TAG concentrations were elevated in HF2 mothers compared to pre-gravidity (e0) and early term (e7) (Figure 2C). Near-term (e20) TAG concentrations were elevated in control and HFG mothers compared to pre-gravidity (e0) and early term (e7); further, near-term (e20) TAG concentrations were elevated in HF1, HF2, and HF3 mothers compared to pre-gravidity (e0), early (e7), and mid-term (e14) (Figure 2C). 

Early term (e7) plasma cholesterol concentrations were reduced in HF2 mothers compared to HF1 mothers (Figure 2D). In HF1 mothers, mid-term (e14) cholesterol concentrations were reduced compared to early (e7) and near-term (e20) (Figure 2D). Near-term (e20) cholesterol concentrations were elevated in HF3 mothers compared to HF2 mothers (Figure 2D). In HF1 mothers, near-term (e20) cholesterol concentrations were elevated compared to pre-gravidity (e0) and mid-term (e14); and in HF2 mothers only relative to mid-term (e14) (Figure 2D). In both HF3 and HFG mothers, near-term (e20) cholesterol concentrations were elevated compared to pre-gravidity (e0), early (e7), and mid-term (e14) (Figure 2D).

### 3.5. Placental Neutral Lipids and Phospholipid Fatty Acids Near Term (e20)

**Figure 3:**
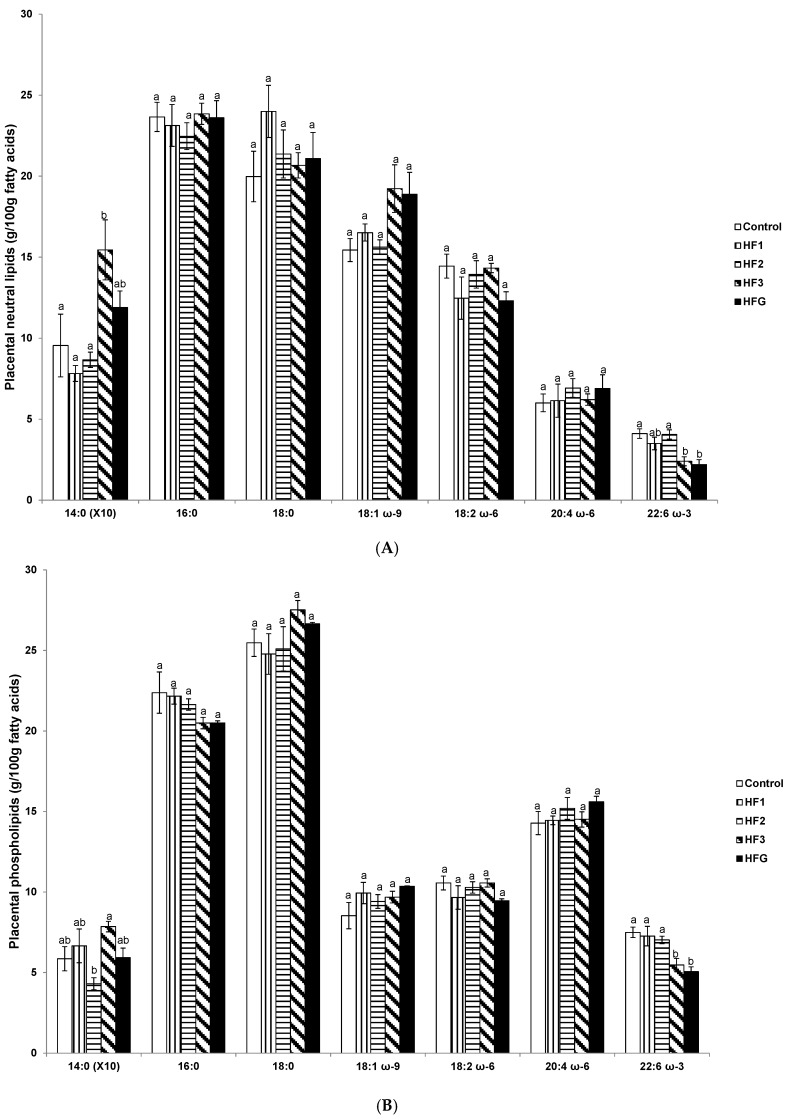
Placental fatty acids. (**A**) Placental neutral fatty acids (g/100 g fatty acids); and (**B**) placental phospholipid fatty acids (g/100 g fatty acids). Numerals refer to the specific week of gestational high fat (HF) maintenance. G, gestation refers to HF maintenance throughout gestation. Values are means ± standard error of the mean (SEM). Different letters reflect significant changes.

In HF3 mothers, placental neutral lipid myristic acid was increased compared to the control, HF1 and HF2 mothers (Figure 3A). In both HF3 and HFG mothers, DHA was reduced compared to control and HF2 mothers (Figure 3A). 

Similarly in HF3 and HFG mothers, placental phospholipid DHA was reduced compared to control, HF1 and HF2 mothers (Figure 3B). Placental phospholipid myristic acid was elevated in HF3 mothers compared to HF2 mothers (Figure 3B).

### 3.6. Plasma Fetal: Maternal Fatty Acids and Fetal and Maternal AA:LA Ratios

**Figure 4:**
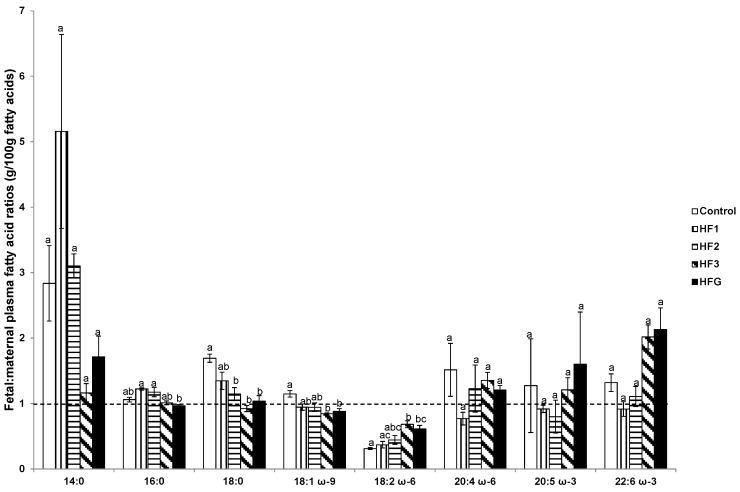
Fetal: maternal ratio of plasma fatty acids (g/100 g fatty acids). Numerals refer to the specific week of gestational high fat (HF) maintenance. G, gestation refers to HF maintenance throughout gestation. Values are means ± standard error of the mean (SEM). Different letters reflect significant changes.

Although we recently reported the FA profiles in fetal plasma [16], we subsequently determined the plasma fetal:maternal ratios of individual FAs. As shown in Figure 4, the fetal:maternal plasma ratio of stearic acid was reduced in HF2, HF3, and HFG mothers compared to control mothers. The fetal:maternal plasma oleic acid ratio in HF3 and HFG mothers was reduced, whereas the LA ratio was elevated relative to control mothers (Figure 4). Fetal:maternal plasma LA ratio was also elevated in HF3 mothers relative to HF1 mothers (Figure 4). Fetal:maternal plasma palmitic acid ratio was reduced in HFG mothers compared to HF1 and HF2 mothers (Figure 4).

Although the fetal:maternal ratio of most FAs did not exceed 1, the DHA ratios in the HF3 and HFG groups were increased ~two-fold, albeit non-significant. The plasma AA:LA ratio in either the fetuses or the mothers did not differ between the different groups, but values were consistently higher in fetuses than in mothers in all groups (Table S4).

### 3.7. Hepatic Fatty Acids

**Figure 5:**
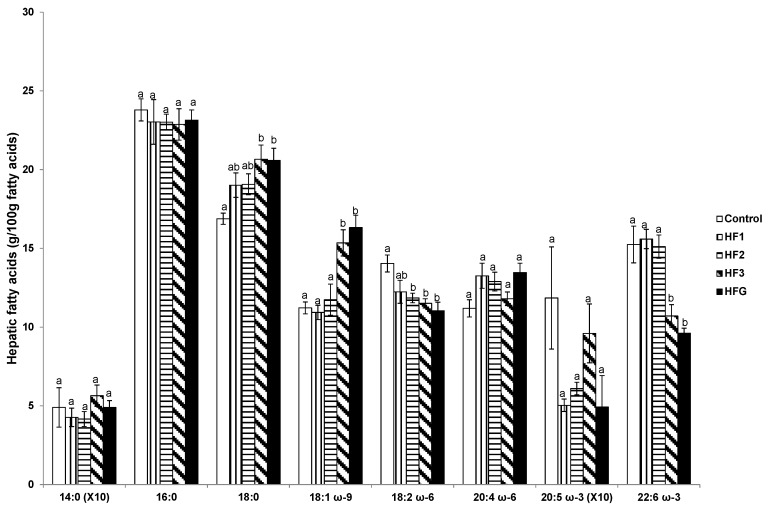
Hepatic fatty acids (g/100 g fatty acids). Numerals refer to the specific week of gestational high fat (HF) maintenance. G, gestation refers to HF maintenance throughout gestation. Values are means ± standard error of the mean (SEM). Different letters reflect significant changes.

Hepatic stearic and oleic acid were elevated in HF3 and HFG mothers whereas LA and DHA were reduced relative to control mothers (Figure 5). Hepatic LA proportions were also reduced in HF2 mothers compared to control mothers (Figure 5). 

### 3.8. Adipose Tissue Fatty Acids

**Figure 6:**
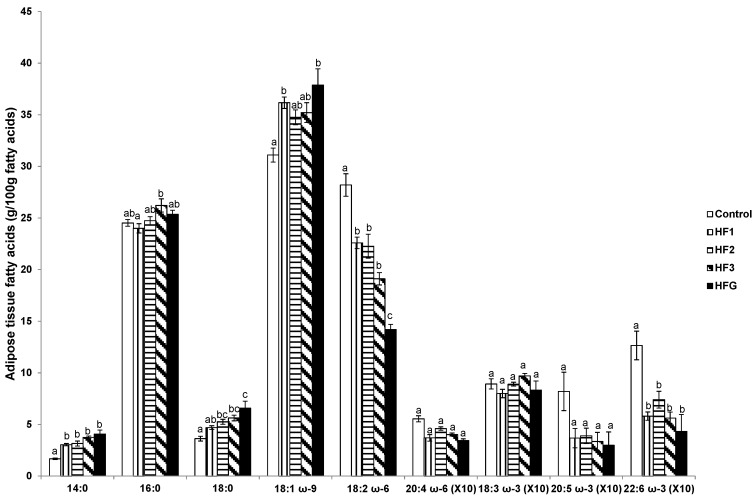
Adipose tissue fatty acids (g/100 g fatty acids). Numerals refer to the specific week of gestational high fat (HF) maintenance. G, gestation refers to HF maintenance throughout gestation. Values are means ± standard error of the mean (SEM). Different letters reflect significant changes.

The proportion of adipose tissue myristic acid was elevated whereas LA and DHA were reduced in HF1, HF2, HF3, and HFG mothers compared to controls (Figure 6). Further, adipose tissue LA was reduced in HFG mothers compared to HF1, HF2, and HF3 mothers (Figure 6). In addition, adipose tissue stearic acid was elevated in HF2, HF3, and HFG mothers relative to control mothers, and in HFG mothers compared to HF1 mothers (Figure 6). Adipose tissue oleic acid was elevated in HF1 and HFG mothers compared to control mothers (Figure 6). Further, adipose tissue palmitic acid was elevated in HF3 relative to HF1 mothers (Figure 6).

## 4. Discussion

The present rodent study sought to determine the effects of HFD administration during specific periods of gestation on maternal lipid profiles, since we recently described its consequences on the availability of FAs to the fetus [16]. The HFD administered was rich in saturated and oleic acids but depleted in LA and DHA relative to the control diet with negligible amounts of αLA. One of the key findings was that the intake of the HFD during the first, second, or third week of gestation or throughout gestation did not modify the intense increase in plasma NEFA that occurs physiologically near-term (in control rats). This observation may reflect an index of adipose tissue lipolysis and, therefore, our data suggest that this pathway is highly augmented during late pregnancy as expected [10]. The main fate of plasma NEFA is the liver [17,18] for partial re-esterification in the synthesis of TAG which are released back into the circulation, and this pathway is also known to be enhanced in late pregnancy in the rat [19,20]. Bearing this in mind, we found that plasma TAG was also greatly increased in all pregnant rats close to term, although the increase seemed to be gradual as it was also evident at the end of mid pregnancy (day 14 of gestation) in those rats maintained on the HFD solely for the second week of pregnancy (HF2) or throughout pregnancy (HFG). This occurred despite unaltered plasma NEFA until near-term in all the groups suggesting that the HFD may increase TAG release from the liver already from mid pregnancy.

The profile of individual FAs in plasma and in the various organs provides insight into the metabolic changes taking place due to the HFD administration during gestation. In agreement with the known direct relationship between FA composition in the diet and maternal adipose tissue in pregnant rats fed different diets [21], the profile of FAs in the adipose tissue of the mothers maintained on the HFD for the different weeks of gestation closely reflected those of the diet. Moreover, there were higher proportions of saturated FAs and oleic acid but lower LA and DHA proportions. However, only the plasma had a decline in DHA in rats fed the HFD for the third week of gestation or throughout gestation suggesting that other organs may compensate for the altered dietary FA profile. This seems to be the case in the liver where an increase in stearic and oleic acid was only evident in those rats fed the HFD for the third week of gestation or throughout gestation. This resonates as the rats were studied at a time when they were maintained on the diet containing high proportions of saturated FAs and oleic acid.

As reported in mice, HF feeding is likely to induce hepatic FA synthesis by chain elongation and subsequent desaturation rather than *de novo* synthesis [22] which may justify the unaltered palmitic acid but enhanced stearic and oleic acid in the livers of the mothers maintained on a HFD during the third week of gestation or throughout gestation, and studied near-term. A different scenario presents with the proportion of LA in the liver which was decreased near-term virtually in all the rats (apart from HF1 mothers) maintained on the HFD for any of the three weeks of gestation. However, the main PUFA derived from LA, AA, did not differ amongst the groups in either plasma or liver. Since the proportion of AA in the diets was very low, this suggested that the conversion of LA to AA was enhanced in those rats fed the HFD. An increase in the proportion of AA was previously reported in the liver of rat mothers, newborn pups and suckling pups that were fed a HFD 10 days prior to mating, throughout pregnancy, and during lactation [23]. These changes could be attributed to the known effects that HFDs have on the expression of genes involved in lipid metabolism that would facilitate LA elongation and desaturation in the synthesis of AA [24]. However, this situation cannot be sustained for DHA since levels of its EFA precursor, αLA, were very low in the diet and therefore the DHA levels mainly depend on its dietary availability which was greatly reduced in the HFD (~10-fold lower) compared to the control diet. The consequence was the consistent decline of DHA availability as demonstrated in the plasma, liver, and adipose tissue of rats fed the HFD for the last week of gestation or throughout gestation.

A similar rationale could be applied to understand the FA profile of placental neutral lipids (involved in lipid storage) and phospholipids (form cell membranes), where the most consistent changes were the decline in the proportions of DHA. The placenta plays a key role in the mother-fetus relationship, maintaining fetal homeostasis through the regulation of nutrient transfer [25] and is involved in materno-fetal exchanges, metabolism, endocrinology, and immune pathways, and is an active component for fetal growth [26]. Although lipogenesis occurs in the rat placenta [27], its rate seems to be slow [28,29] and, therefore, most placental FAs are a result of the balance between those taken up from maternal plasma and those released to the fetus. In the present study, DHA was the only FA that was consistently reduced in both placental neutral lipids and phospholipids in rats fed the HFD for the third week of gestation and throughout gestation. This change reflected the decrease of this PUFA in plasma and therefore may be a consequence of its limited uptake by the placenta from maternal circulation. The placenta is known to preferentially transfer PUFA, such as DHA, due to their absolute requirement for brain and retina development [30]. Further, the reduced placental phospholipid DHA profiles in rats maintained on the HFD for the third week of gestation or throughout gestation may also result in reduced transfer of this PUFA to their fetuses which may potentially stunt fetal brain and retina development. Interestingly, the fetal:maternal DHA ratio increased ~two-fold in rats maintained on the HFD for the third week of gestation or throughout gestation although the change *versus* the one value was not statistically significant. This finding resonates with the known magnification of this specific FA in the fetus in relation to the mother indicating a higher proportional placental transfer of DHA relative to other FAs [31], although absolute concentrations are lower in the fetus than in the mother under different dietary regimens [32] as also shown in humans [3]. 

The metabolic state and nutrition of mothers during pregnancy has developmental programming consequences for their progeny. PUFA dietary content, particularly a more balanced ω-6 to ω-3 ratio, may be a key dietary variable in the developmental programming of cardiometabolic function in adult offspring [33]. In rodent studies, the ω-6 to ω-3 FA ratio in the maternal diet may impact bone parameters and therefore indirectly also the body weight [34]. Modulation of dietary ω-6 to ω-3 ratio and/or early leptin levels may also have long-term effects on later metabolic parameters [35]. Increased maternal intake of ω-3 FAs led to a decreased growth rate, reduced adipose tissue mass, lower serum leptin concentrations [36], reduced fat accretion, and reduced the age-related decline in insulin sensitivity in progeny [37]. The reduced DHA in mothers maintained on a HFD for the third week of gestation or throughout gestation in the plasma, liver, adipose tissue, and placenta may predispose their offspring to metabolic disease.

In humans, DHA supplementation during normal pregnancy was associated with lower infant ponderal index at birth and decreased umbilical cord insulin concentrations compared to infants from mothers consuming placebos [38]. Higher cord plasma DHA concentrations and ω-3 to ω-6 PUFA ratios were associated with improved fetal insulin sensitivity, whereas cord plasma saturated FAs (namely stearic acid and arachidic acid (C20:0)) were negatively correlated with fetal insulin sensitivity, suggesting a positive impact of certain ω-3 FAs and a negative impact of saturated FAs on fetal insulin sensitivity [39]. This may be explained by PUFAs constituting important structural elements of cell membranes and, concomitant with their eicosanoid products, they also modulate gene expression [35]. In our study, in rats maintained on a HFD for the third week of gestation, plasma myristic and stearic acid proportions were elevated, whereas DHA proportions were reduced in both mothers maintained on a HFD during the third week of gestation or throughout gestation, which likely contributes to insulin insensitivity in their fetal offspring. Indeed, fetuses exposed to a HFD for the third week of fetal life were heaviest concomitant with elevated glycemia and insulin resistance [16] reflecting ,insulin insensitivity.

Unfortunately, the mechanisms involved cannot be derived from our data, although other studies provide some insight. Studies in pregnant mice reported that a HFD causes marked up-regulation of placental transport of specific nutrients, such as glucose and neutral amino acid transport [13]. Thus, since DHA is essential for normal fetal and neonatal growth and development [5,40], its deficiency in the HFD may enhance its placental transfer. The impact of dietary ω-3 on placental function is well recognized [41]. Another consideration is that an ω-3 deficient condition, as induced by the HF administration during gestation, could up-regulate the fetal synthesis of DHA from its EFA precursor, αLA. The near-term rat fetus has the capacity to synthesize DHA from αLA [42,43] via different desaturases and elongases [44,45,46,47]. Moreover in the adult rat, it was shown that an ω-3 deficient diet up-regulates the hepatic expression and activity of delta-6-desaturase [48] and the synthesis of DHA from αLA is enhanced by diets low in PUFA [49]. Additional studies are required to determine which of these mechanisms are in effect to justify the trend of the increase in the proportion of DHA in fetal plasma relative to their mothers under conditions of HF feeding during late pregnancy.

## 5. Conclusions

Mothers maintained on a HFD for the final week of gestation or throughout gestation had reduced DHA in plasma, liver, adipose tissue, and placenta. Altered circulating and hepatic FA profiles, particularly saturated FA elevation, and reduced key ω-3 and ω-6 PUFA, coinciding with the compromised maternal metabolic state of pregnancy, reflects altered lipid metabolism that may have adverse health outcomes. Low DHA maternal availability in rats fed the HFD may up-regulate its placental transfer or stimulate its fetal synthesis from its EFA precursor thereby allowing the maintenance of a higher proportion in fetal plasma relative to maternal plasma. Therefore, maternal intake of a HFD during the critical stage of peak fetal development should be avoided for stabilizing lipid profiles to promote healthy outcomes in mothers and their fetuses.

## Supplementary Materials

Supplementary materials can be accessed at: http://www.mdpi.com/2072-6643/8/1/25/s1.
