# Supplementary material for: High Fat Diet Administration during Specific Periods of Pregnancy Alters Maternal Fatty Acid Profiles in the Near-Term Rat
Source: Nutrients. 2016 Jan 4;8(1):25. doi: 10.3390/nu8010025 (PMC4728639; doi:10.3390/nu8010025)
Supplement: Supplementary file 1 [file nutrients-08-00025-s001.docx]

Supplementary Materials: High Fat Diet Administration during Specific Periods of
Pregnancy Alters Maternal Fatty Acid Profiles
in the Near-Term Rat

Marlon E. Cerf and Emilio Herrera

**Table S1.** Macronutrient profiles.

| **Macronutrient** | **Control Diet** | **HFD** |
| --- | --- | --- |
| Carbohydrate | 75% | 46% |
| Protein | 15% | 14% |
| Fat | 10% | 40% |
| Kcal/g | 2.6 | 2.06 |

HFD, high fat diet. The control diet was a standard commercial laboratory rodent diet. The HFD predominantly comprised saturated FA (myristic, palmitic and stearic acid) and the mono-unsaturated FA, oleic acid, derived from animal fat with carbohydrates mainly derived from starch to mimic a westernized diet.

**Table S2.** Anthropometry.

|  | **Control** | **HF1** | **HF2** | **HF3** | **HFG** |
| --- | --- | --- | --- | --- | --- |
| **Food intake (g)** | 405.9 ± 9.95 | 446.1 ± 16.02 | 431.2 ± 22.32 | 473.3 ± 8.23 | 615.9 ± 33.12 *^,†,‡,§^ |
| **Weight (g)** | 294 ± 12.70 | 300 ± 9.56 | 294 ± 9.65 | 290 ± 7.49 | 322 ± 13.95 |
| **Placenta weight (g)** | 0.86 ± 0.04 | 0.72 ± 0.03 | 0.76 ± 0.03 | 0.75 ± 0.04 | 0.75 ± 0.03 |
| **Adjusted placenta weight** | 26.58 ± 1.20 | 23.40 ± 1.39 | 22.79 ± 0.86 | 19.72 ± 1.11 * | 23.65 ± 1.01 |
| **Conceptus weight (g)** | 55.90 ± 3.64 | 56.28 ± 8.01 | 55.58 ± 4.28 | 50.91 ± 7.65 | 58.43 ± 7.22 |
| **Adjusted conceptus weight** | 19.54 ± 0.38 | 19.02 ± 2.41 | 18.93 ± 1.31 | 17.53 ± 2.49 | 17.99 ± 1.63 |
| **Litter size** | 10.33 ± 0.88 | 10.00 ± 1.58 | 9.83 ± 0.48 | 8.50 ± 1.19 | 10.50 ± 1.56 |
| **Liver weight (g)** | 1.24 ± 0.13 | 0.91 ± 0.12 | 0.67 ± 0.16 | 0.98 ± 0.13 | 0.99 ± 0.09 |
| **Adjusted liver weight** | 0.42 ± 0.03 | 0.31 ± 0.05 | 0.23 ± 0.05 | 0.34 ± 0.04 | 0.31 ± 0.04 |
| **Adipose tissue weight (g)** | 0.47 ± 0.07 | 0.84 ± 0.17 | 0.59 ± 0.17 | 0.74 ± 0.06 | 0.90 ± 0.12 |
| **Adjusted adipose tissue weight** | 0.16 ± 0.02 | 0.29 ± 0.06 | 0.20 ± 0.05 | 0.26 ± 0.02 | 0.28 ± 0.03 |

The mothers were maintained on a high fat diet (HFD) for either the first week (HF1), second week (HF2), third week (HF3), or all 3 weeks (HFG) of gestation. Ajusted organ weight = organ weight/body weight × 100. Values are means ± SEM. *p* < 0.05 * *vs.* control, ^†^ *vs.* HF1, ^‡^ *vs.* HF2, ^§^ *vs.* HF3.

**Table S3.** Glucose and insulin concentrations and HOMA-insulin resistance.

|  | **Control** | **HF1** | **HF2** | **HF3** | **HFG** |
| --- | --- | --- | --- | --- | --- |
| **Glucose (mmol/L)** | 8.6 ± 0.65 | 8.3 ± 0.70 | 7.7 ± 1.19 | 8.5 ± 1.28 | 9.7 ± 0.80 |
| **Insulin (pM)** | 246.9 ± 118.8 | 367.8 ± 103.9 | 376.3 ± 84.39 | 189.6 ± 57.14 | 374.6 ± 110.3 |
| **HOMA-IR** | 103.9 ± 57.36 | 127.8 ± 25.99 | 134.1 ± 39.59 | 80.99 ± 36.34 | 155.9 ± 37.71 |

The mothers were maintained on a high fat diet (HFD) for either the first week (HF1), second week (HF2), third week (HF3), or all 3 weeks (HFG) of gestation. Values are means ± SEM.

**Table S4.** Plasma fetal and maternal AA:LA ratio (g/100 g fatty acids).

|  | **Fetus** | **Mother** | ***p*** |
| --- | --- | --- | --- |
| **Control** | 0.73 ± 0.05 | 0.30 ± 0.08 | 0.0048 |
| **HF1** | 0.76 ± 0.11 | 0.37 ± 0.05 | 0.0305 |
| **HF2** | 0.83 ± 0.09 | 0.42 ± 0.10 | 0.0255 |
| **HF3** | 0.70 ± 0.03 | 0.36 ± 0.02 | 0.0093 |
| **HFG** | 0.85 ± 0.04 | 0.44 ± 0.02 | <0.0001 |

The fetuses and mothers were maintained on a high fat diet (HFD) for either the first week (HF1), second week (HF2), third week (HF3), or all 3 weeks (HFG) of gestation. Values are means ± SEM.
